# The Clinical Use of Myo-Inositol in IVF-ET: A Position Statement from the Experts Group on Inositol in Basic and Clinical Research and on PCOS (EGOI-PCOS), the Polish Society of Andrology, and the International Scientific Association for the Support and Development of Medical Technologies

**DOI:** 10.3390/jcm14020558

**Published:** 2025-01-16

**Authors:** Artur Wdowiak, Szymon Bakalczuk, Michał Filip, Antonio Simone Laganà, Vittorio Unfer

**Affiliations:** 1 Obstetrics and Gynecology Faculty of Health Sciences, Medical University of Lublin, 20-081 Lublin, Poland; wdowiakartur@gmail.com; 2 The Experts Group on Inositol in Basic and Clinical Research, and on PCOS (EGOI-PCOS), 00161 Rome, Italy; antoniosimone.lagana@unipa.it; 3 University Clinical Hospital No. 1 in Lublin, 20-400 Lublin, Poland; szymonbakalczuk@o2.pl; 4 Polish Society of Andrology, 21-030 Lublin, Poland; 5 Department of Obstetrics and Pathology of Pregnancy, Faculty of Medicine, Medical University of Lublin, 20-081 Lublin, Poland; michal.a.filip@gmail.com; 6 International Scientific Association for the Support and Development of Medical Technologies, 20-012 Lublin, Poland; 7 Unit of Obstetrics and Gynecology, “Paolo Giaccone” Hospital, Department of Health Promotion, Mother and Child Care, Internal Medicine and Medical Specialties (PROMISE), University of Palermo, 90127 Palermo, Italy; 8 Department of Gynecology and Obstetrics, UniCamillus–Saint Camillus International University of Health Sciences, 00131 Rome, Italy

**Keywords:** myo-inositol, PCOS, IVF, ovarian stimulation, gonadotropins

## Abstract

**Background:** Myo-inositol plays a vital role in human health, functioning as a second messenger of FSH and facilitating the transport of glucose into the cell. Consequently, myo-inositol is regularly utilized in the treatment of polycystic ovary syndrome (PCOS), wherein it acts upon metabolic factors, improving insulin sensitivity and reducing total androgen levels. Patients with PCOS frequently suffer from infertility; thus, the use of myo-inositol has been explored in improving assistive reproductive technique (ART) procedures. This is by no means limited to patients with PCOS, as inositol has found applications in non-PCOS patient groups in addition to in male factor infertility. This joint statement from the Experts Group on Inositol in Basic and Clinical Research and on PCOS (EGOI-PCOS), the Polish Society of Andrology, and the International Scientific Association for the Support and Development of Medical Technologies discusses the latest evidence on this topic, with the aim of interrogating whether myo-inositol could be implemented in everyday ART patient care. **Methods:** The authors conducted a narrative review performed via an independent literature search between July and August 2024, using the search platforms PubMed, Web of Science, and Google Scholar. **Results:** In both non-PCOS and PCOS populations seeking IVF care, MI supplementation prior to ovarian stimulation may positively affect gonadotropin use and duration, oocyte and embryo quality, fertilization, and clinical pregnancy rates. **Conclusions:** This position statement recommends that myo-inositol be considered as a potential pretreatment strategy prior to ovarian hyperstimulation with gonadotropins.

## 1. Introduction

Since its inception, in vitro fertilization (IVF) has revolutionized how humanity considers and addresses human reproduction. Infertility affects approximately 10% of couples, such that the reliance upon IVF is increasing, with nearly 5% of children in Europe born via IVF [1]. The reliance upon IVF is more pronounced in developed countries, where women regularly wait until their thirties to pursue pregnancy. Furthermore, IVF allows same sex couples to start a family, as previously, it would not have been possible without the use of adoption. Within the context of IVF, there has been a growing movement to consider access to fertility as a human right [2,3], demonstrating the importance of fertility care to modern society.

Despite IVF providing enormous assistance to couples seeking pregnancy, it is not without issues. Ovarian hyperstimulation syndrome (OHSS) is a potentially life-threatening complication of ovarian stimulation, an integral part of IVF procedures [4]. Mild symptoms of OHSS include digestive issues such as nausea and vomiting, while in more severe to critical cases, symptoms include intense abdominal pain, ascites, hypovolemia, an increased risk of thromboembolism, pleural effusion or even renal failure, and death [5]. The probability of severe OHSS complications was reported by the World Health Organization (WHO) to be between 0.2 and 1%; therefore, it is paramount to continue research into improving the safety profile of stimulation procedures.

IVF response rates are not equal across all patients, and poor responders may face several IVF-ET cycles without a successful birth. This can put a psychological and economic burden on couples seeking fertility care. Furthermore, the risk of multiple gestations is increased with the use of IVF, as reflected by the higher proportion of twins and triplets born via IVF in comparison to in the general population [6]. Lastly, the use of IVF has been demonstrated to increase pregnancy complications including hypertension, preterm delivery, and gestational diabetes (GDM) [7]. Consequently, it is apparent that current IVF protocols need constant development in order to reduce such risks.

One natural molecule that has been utilized to reduce such IVF complications is myo-inositol (MI). Inositol is a carboxylic polyalcohol with several stereoisomers, of which MI is the most common. MI is a conditional nutrient, with part of the daily recommended dose of MI being able to be synthesized within the body [8], and the rest being obtained from MI-rich foods including nuts, fruits, grains, and beans [9]. MI supplementation has shown success in the field of hyperandrogenic PCOS, wherein it functions as an insulin sensitizer in patients with metabolic abnormalities [10]. MI performs this function by facilitating the transportation of glucose into the cell via the GLUT-4 pathway [11]. Furthermore, MI plays a role in ovarian function by acting as a second messenger to FSH [12], thus modulating the production of the FSH-dependent anti-Mullerian hormone (AMH) and playing a role in oocyte maturation [13].

MI supplementation has also been demonstrated to enhance the efficacy and efficiency of IVF procedures, reducing the required doses of gonadotropin used during ovarian stimulation and potentially increasing oocyte and embryo quality, and the implantation rate. In 2024, the Italian Society of Human Reproduction advocated for the inclusion of MI in IVF protocols [14]; however, this opinion was not shared by the International Guidelines for PCOS, which cited limited and inclusive evidence [15]. This position statement from the Experts Group on Inositol in Basic and Clinical Research and on PCOS (EGOI-PCOS), the Polish Society of Andrology, and the International Scientific Association for the Support and Development of Medical Technologies delves into the potential for MI use in general IVF practice in patients both with and without PCOS in order to improve pregnancy outcomes.

## 2. Methodology

The authors performed an independent literature search, with search terms including the following: myo-inositol, inositol, d-chiro-inositol, in vitro fertilization, polycystic ovary syndrome, intracytoplasmic sperm injection, and male factor infertility. The publication search was conducted using the following databases: PubMed, Web of Science, and Google Scholar. The literature search was conducted between July and August 2024.

## 3. MI in IVF Care for Patients Without PCOS

Due to its role in FSH signaling, MI has been investigated as a key molecule in ovarian function and fertility care. While no specific studies have evaluated the safety of MI in IVF care and pregnancy, the molecule is on the Generally Recognized As Safe (GRAS) list and demonstrates only minor gastrointestinal effects at dosages beyond 12 g/day, which falls outside the recommended daily dose [16]. The use of MI as a pretreatment strategy prior to IVF care has been demonstrated to improve pregnancy outcomes in women with infertility. In a prospective study, 46 patients with infertility who had previously undergone unsuccessful IVF cycles were treated with a daily combination of MI (4 g) in combination with melatonin (3 mg) (Table 1) [17]. After supplementation, the number of mature oocytes (*p* = 0.02), in addition to the number (*p =* 0.01) and the quality of embryos transferred (*p =* 0.01), significantly increased in comparison to the first IVF treatment cycle. Building upon this work, Seyedoshohadaei et al. conducted a double-blind randomized controlled trial in 70 women with infertility who received either MI (4 g/day) with folic acid (400 mg/day) or folic acid alone over a period of 2 months [18]. This study demonstrated a significant increase in the number of oocytes retrieved following MI supplementation, in addition to improved oocyte and embryo quality (*p* = 0.04), with a better overall average ART outcome for the study group.

MI has been shown to be effective in poor responders to IVF. The definition of poor responders was standardized in 2011 with use of the Bologna criteria, which states that poor responders must present with at least of two of the following: advanced maternal age or other risk factors for poor IVF response, previous poor response to IVF, and/or an abnormal ovarian reserve test [19]. Poor responders often undergo numerous failed cycles of IVF, which may lead to an eventual cessation of IVF care. In 2015 Caprio et al. conducted a pilot study which compared a three-month pretreatment of 4 g of MI in combination with 400 µg of folic acid and folic acid alone, prior to ovarian stimulation, in 77 poor responders [20]. Compared to the folic acid control, MI supplementation resulted in a significant reduction in the units of rFSH required for ovarian stimulation (*p* = 0.0004), and an improvement in oocyte quality (*p* = 0.001) and in the ovarian sensitivity index (OSI) (*p* < 0.05), defined as the total administered rFSH dose divided by the number of oocytes retrieved at ovum pick-up. A larger randomized control trial pretreated patients with MI and folic acid at the same concentrations, however only for one month [21]. Consequently, similar results were not observed between the two studies, with no significant difference observed between the study and control group in terms of gonadotropin use and the quality of oocytes. However, this study did observe a significant increase in embryo quality and fertilization rate in the MI-supplemented group. A final trial of 60 poor responders followed the same treatment regimen as the Caprio study [22]. This study also observed a significant reduction in the required units of gonadotropins, in addition to a significantly increased fertilization rate and OSI (*p* < 0.05).

D-chiro-inositol (DCI), the most common stereoisomer of MI, is known to act as an insulin sensitizer, primarily involved in facilitating glycogen storage and inducing white-adipose-to-brown-adipose tissue differentiation [23]. Consequently, DCI has been investigated as a potential therapeutic for PCOS and other metabolic disorders such as obesity. Given the chemical similarities between the two isomers, both are routinely investigated for the same therapeutic applications, despite their notably different roles in signaling pathways. Recently, concern has been raised regarding the use of DCI at high doses, as it has been demonstrated to inhibit aromatase transcription leading to increased testosterone levels, thus introducing risks to conception and pregnancy [24]. It was posited by Ravanos et al. that due to the potential harmful role of DCI in embryo development, the ratio of MI:DCI may serve as a useful biomarker for successful embryo implantation and pregnancy [25]. Specifically, the follicular fluid of eight egg donors undergoing IVF was assessed for blastocyte quality (according to Gardner’s grading system [26]) and the MI/DCI ratio. It was observed that grade 4 and grade 3 embryos had a higher MI ratio than those graded 2 or 1 (grade 4–3: 66.19 [53.82–142.00] vs. grade 2–1 49.54 [47.91–55.56]), suggesting a correlation between blastocyte quality and the MI:DCI ratio.

**Table 1:** Summary of studies of myo-inositol in patients without PCOS receiving IVF care.

Reference	Study Type	Number of Patients	Treatment Type and Length	Primary Findings
Unfer (2011) [17]	Prospective longitudinal cohort study	46 women undergoing IVF who had previously undergone failed IVF cycles due to poor oocyte quality.	MI (2 g in the morning, 2 g in the evening) + melatonin (3 mg in the evening) daily for three months.	Significant increase in mature oocytes, fertilization rate, and quality and quantity of embryos transferred compared to the previous IVF cycle.
Seyedoshohadaei (2022) [18]	Double-blind randomized control trial	70 infertile women referred for fertility treatment. n = 36 study group; n = 34 placebo.	Study group: MI (2 g) + folic acid (200 µg) sachet twice daily. Control group: folic acid (200 µg) twice daily for 2 months.	Significant increase in mean numbers of oocytes, oocyte quality, clinical pregnancy, and live birth rates in the treatment group versus controls.
Caprio (2015) [20]	Prospective controlled observational trial	76 poor responders according to the Bologna criteria, undergoing IVF ICSI care. n = 38 study group; n = 38 control group.	Study group: MI (4 g) + folic acid (400 µg) daily. Control group: folic acid (400 µg) daily for 1 month prior to COH.	Significantly reduced total rFSH units required for COH and significantly higher OSI, and M2 oocytes in the study group versus the control. No significant difference in estradiol levels between the two groups.
Nazari (2020) [21]	Open label randomized controlled observational trial	112 poor responders according to the Bologna criteria, undergoing IVF ICSI care. n = 56 study group; n = 56 control group.	Study group: MI (4 g) + folic acid (400 µg) daily. Control group: folic acid (400 µg) daily for 3 months prior to COH.	No significant difference between the groups across the total number of gonadotropins used, OSI, total oocytes received, implantation and pregnancy rates, and the number of mature oocytes. Significantly increased grade A embryos and fertilization rate in the study group versus the control.
Mohammadi (2021) [22]	Double-blind randomized control trial	60 poor responders according to the Bologna criteria, undergoing IVF ICSI care. n = 30 study group; n = 30 control group.	Study group: MI (4 g) + folic acid (400 µg) daily. Control group: folic acid (400 µg) daily for 3 months prior to COH.	No significant difference in the number of oocytes retrieved, embryos transferred, and clinical pregnancy between the two groups. Significantly higher OSI and fertilization rate and significantly reduced total gonadotropin units required for COH in the study group versus the control.

COH: controlled ovarian hyperstimulation; ICSI: intracytoplasmic sperm injection; IVF: in vitro fertilization; MI: myo-inositol; OSI: ovarian sensitivity index

## 4. MI in Patients with PCOS Receiving IVF Care

PCOS represents one the main contributors to infertility in women, representing 80% of anovulatory infertility cases [27]. In general practice, MI is utilized in a similar manner to metformin, acting as an insulin sensitizer in PCOS, reducing the hyperandrogenism commonly associated with metabolic disturbances such as insulin resistance. In addition, its role as a second messenger for FSH aids in the restoration of a regular ovulatory cycle, which is required for a successful IVF cycle.

Both MI and metformin have been utilized as pretreatments for patients with PCOS undergoing IVF procedures. Raffone et al. studied the effects of insulin sensitizers both alone and in combination for the induction of ovulation in women with PCOS (Table 2). [28]. Specifically, ovulation was achieved in 50% of patients treated with metformin alone, resulting in an 18.3% pregnancy rate. A greater response was observed in the MI group, with 65% of patients achieving ovulation and a 30% pregnancy rate. Patients who did not achieve pregnancy after 6 months of therapy underwent ovarian stimulation with rFSH in combination with either metformin or MI, whereby MI was shown to be more effective (28.9% pregnancy rate for MI vs. 26.1% pregnancy rate for metformin). The use of MI and metformin in combination was compared to MI alone in a prospective clinical study in 120 infertile insulin-resistant women with PCOS [29]. The women were divided into two equal groups and pretreated prior to ovarian stimulation over a period of three months. The MI and metformin group had significantly increased menstrual cycle regularity and live birth rate, in addition to decreased HOMA-IR, versus the metformin group. A further double-blind randomized controlled trial compared patients with PCOS who had received pretreatment with either MI (2 g, twice daily) or metformin (850 mg, twice daily) [30]. Metrics such as incidences of OHSS, duration and dose of gonadotropins, implantation rate, and the number of embryos available for freezing were not significantly different between the two groups. In contrast, the clinical and cumulative pregnancy rate, and spontaneous pregnancy prior to ovarian stimulation were significantly higher in the MI group. The difference in these studies underscores that further large RCTs are required to confirm the differences between MI and metformin in ART procedures for PCOS populations.

In a study by Özay et al. [31]., 196 patients were split between two groups and underwent controlled ovarian hyperstimulation (COH) with or without MI supplementation. In this study, MI significantly reduced the amount of rFSH used and regulated menstrual cyclicity, in addition to significantly improving the clinical pregnancy rate vs. the control group (18.6% in study group vs. 12.2% in control group). In concordance with these results, a prospective randomized trial of patients with PCOS receiving either MI (4 g per day) in combination with folic acid (400 µg) or a placebo observed increased fertilization rates and better embryo quality in the MI group in comparison to the placebo group [32]. Furthermore, the MI group had a reduced stimulation period (9.7 ± 3.3 days) vs. the control group (11.2 ± 1.8 days) and reduced rFSH usage (study group: 1750 rFSH units vs. control: 1850 rFSH units).

A recent in silico study evaluated the potential for MI to reduce gonadotropin use in IVF patients, reducing the probability of OHSS in addition to potentially resulting in financial savings for state healthcare systems [33]. This study used a Markov model to simulate IVF procedures for 100,000 virtual patients undergoing ovarian stimulation with rFSH either alone or in combination with MI. The data input into the model were taken from previous publications and clinical experience. The rFSH + MI group demonstrated an improved rate of ongoing pregnancy at 12 gestational weeks in comparison to rFSH alone (rFSH + MI 0.38 ± 0.04 vs. rFSH 0.36 ± 0.06, *p* < 0.0001). This study also demonstrated a reduction in the cost of IVF procedures and the overall cost of a successful pregnancy, and both results stemmed from the number of rFSH vials used in the stimulation protocol.

In a meta-analysis of seven trials and 935 women, Zheng et al. investigated the efficacy of MI in studies including infertile patients with and without PCOS. In this analysis, MI supplementation led to significant improvements in pregnancy rate (*p* = 0.03), abortion rate (*p* = 0.0006), the proportion of grade I embryos (*p* = 0.02), and the required units of stimulation drugs (*p* = 0.02) [34]. An additional meta-analysis of eight studies incorporating 912 women investigated the amount of gonadotropin units required in combination with MI versus a control treatment. This analysis included women with and without PCOS and observed a significant reduction in the required units of gonadotropins (*p* < 0.00001) required for stimulation protocols in the PCOS group. Additionally, a reduction was also observed in the non-PCOS group; however, this change was not significant [35].

DCI is thought to be poorly suited to high-dose supplementation during ART procedures; however, its use in a 40:1 MI:DCI ratio has shown some success. This 40:1 ratio reflects the physiological ratio found in blood [36] and makes use of the insulin-sensitizing effect of both molecules, whilst keeping the dose of DCI low so as not to elevate androgen concentrations. The combination of MI and DCI is of particular interest in patients with PCOS who often present as obese and with insulin resistance, which may worsen the hyperandrogenism seen in these patients, thus leading to a higher failure rate of ART procedures [37]. In a randomized prospective controlled trial, patients received either MI:DCI or DCI alone, with improved embryo and oocyte quality and pregnancy rates in the combination group vs. in the group treated with DCI alone [38]. The supplementation of MI/DCI at the 40:1 ratio requires further study, as it should be established whether the combination of these stereoisomers is superior to the use of MI alone. As obesity is a common factor in reduced fertility, the combined insulin-sensitizing effect of these molecules may be particularly effective for obese women with PCOS seeking fertility care and should be investigated more thoroughly.

**Table 2:** Summary of studies of myo-inositol in patients with PCOS receiving IVF care.

Reference	Study Type	Number of Patients	Treatment Type and Length	Primary Findings
Raffone (2010) [28]	Prospective randomized trial	120 anovulatory women with PCOS. n = 60 metformin group; n = 60 MI + folic acid group.	Metformin group: metformin (1500 mg) daily for 6 months. MI + folic acid group: MI (4 g) + folic acid (400 µg) daily for 6 months.	A total of 55% of patients within the metformin group achieved spontaneous ovulation, with those who did not being treated with rFSH + metformin. The total pregnancy rate in the metformin cohort was 26.1%. A total of 65% of patients within the MI + folic acid group achieved spontaneous ovulation, while those who did not ovulate were treated with rFSH + MI + folic acid. The total pregnancy rate in the MI + folic acid cohort was 48.4%.
Agrawal (2019) [29]	Randomized controlled trial	120 infertile women with PCOS. n = 60 group I; n = 60 group II.	Group I: metformin (500 mg) + MI (600 mg) thrice daily for 3 months. Group II: metformin (500 mg) thrice daily for 3 months. Those who did not conceive were given three cycles of ovulation induction + intrauterine insemination.	Significant improvements were observed in menstrual regularity and HOMA-IR in group I versus group II. Live birth rate was also significantly increased in group I versus group II.
Rajasekaran (2022) [30]	Double-blind randomized controlled trial	102 infertile women with PCOS recruited for IVF care. n = 50 MI group; n = 52 metformin group.	MI group: MI (2 g) twice daily for 3 months. Metformin group: metformin (850 mg) twice daily for 3 months. Following therapy, both groups began COH.	A significantly higher clinical pregnancy rate, cumulative pregnancy rate, spontaneous conception rate, fertilization rate, and number of good-quality embryos were observed in the MI group. No difference was observed in either group for the incidence of OHSS, duration of stimulation, gonadotropin units required, number and quality of oocytes retrieved, implantation rate, and number of good-quality embryos for freezing.
Özay (2017) [31]	Prospective randomized controlled trial	196 infertile women with PCOS recruited for IVF care. n = 98 study group; n = 98 control group.	Study group: MI (4 g) + folic acid (400 µg) daily 3 months prior to and during COH. Control group: folic acid (400 µg) during COH.	Significantly less gonadotropin use and significantly higher clinical pregnancy rates were observed in the study group versus the control.
Lesoine (2016) [32]	Prospective randomized controlled trial	29 women with PCOS undergoing IVF care. n = 14 study group; n = 15 control group.	Study group: MI (4 g) + folic acid (400 µg) daily 2 months prior to COH. Control group: placebo.	A significantly higher fertilization rate and embryo quality was observed for the study group versus the placebo. Furthermore, a reduced number of gonadotropins was used for COH in the study group versus the placebo group; however, this was not significant.
Colazingari (2013) [38]	Prospective randomized trial	100 women with PCOS undergoing IVF care. n = 47 group I; n = 53 group II.	Group I: MI (550 mg) + DCI (13.8 mg) twice daily for 3 months. Group II: DCI (500 mg) twice daily for three months. Treatment continued up to COH and throughout pregnancy.	Significantly higher oocyte and embryo quality, in addition to pregnancy rates, were observed in group I versus group II.

COH: controlled ovarian hyperstimulation; DCI: d-chiro-inositol; HOMA-IR: Homeostatic Model Assessment for Insulin Resistance; IVF: in vitro fertilization; MI: myo-inositol; OHSS: ovarian hyperstimulation syndrome; PCOS: polycystic ovary syndrome.

## 5. MI in Male Factor Infertility

Male factor infertility is one of leading obstacles for couples who are experiencing problems when trying to conceive, and it accounts for approximately 20–70% of all infertility cases [39]. Typically, male factor infertility is characterized by conditions such as oligozoospermia (reduced sperm movement), asthenozoospermia, and teratozoospermia, all three of which are described as oligoasthenoteratozoospermia (OAT) [40]. While the cause of male factor infertility is often not easily explained, several genetic and environmental causes are known to contribute to this condition [41].

Although typically associated with female fertility care, MI has garnered interest as a potential treatment in male fertility. Specifically, MI has been demonstrated to reduce the presence of amorphous fibrosis, which is commonly present on the sperm of patients with oligozoospermia [42]. Furthermore, MI can regulate the osmotic properties of seminal fluid, as low osmolarity may reduce sperm motility. In this context, a randomized clinical trial of 37 patients with oligoasthenospermia demonstrated that the incubation of sperm with MI (2 mg/mL) resulted in a significant increase in sperm motility (*p* = 0.0001) [43]. This increase in sperm motility resulted in a two-fold increase in pregnancy in comparison to the control group. Numerous studies have examined the use of MI in male factor infertility. One double-blind RCT investigated the effect of MI in combination with folic acid in men with idiopathic infertility, resulting in a significantly increased acrosome-reacted spermatozoa, sperm concentration, total sperm count, and sperm progressive motility compared to folic acid alone (*p* < 0.5) (Table 3) [44]. In addition, serum gonadotropin and inhibin B levels were rebalanced following MI supplementation, with a reduction in FSH and an increase in inhibin B. This is notable as the balance between FSH and inhibin B is thought to be essential for healthy spermiogenesis [45]. In a prospective longitudinal study, Montanino Oliva investigated the use of MI (1 g) as part of a multi-nutrient in men with asthenospermia and metabolic syndrome [46]. The described supplementation regime resulted in an improved metabolic profile of the participants, in addition to significantly improved sperm concentration, motility, and normal morphology (*p* < 0.001). Interestingly, the authors report significantly increased free and total testosterone and sex hormone-binding globulin (SHBG) levels. The explanation for these hormonal changes is not known; however, the authors speculate that this may be an indirect effect resulting from metabolic changes in the patient cohort.

An improvement in sperm parameters may result in improved fertilization rates during intracytoplasmic sperm injection (ICSI) cycles. In a randomized prospective trial, Rubino et al. pretreated spermatozoa with either MI (2 g) or a placebo to observe the effect of MI supplementation on fertilization rates [47]. Specifically, significant increases were observed in fertilization rate (*p* = 0.002) and in the percentages of grade A embryos (*p* = 0.019), suggesting that MI may improve culture conditions in ART protocols.

A further potential application of MI is as part of sperm cryopreservation protocols. Cryopreservation and sperm banking represent the most common uses for cryobiology, allowing the preservation of fertility in men who have lost fertility either temporarily or permanently [48]. While it is fundamental in modern-day ART, cryopreservation can damage mitochondria and genetic integrity in sperm cells [49]. Furthermore, cryopreservation increases the presence of reactive oxygen species (ROS), which can damage the mitochondrial membrane and lead to reduced sperm viability, motility, and morphology [50]. In this context, MI has been theorized to offer a cryoprotective effect via the activation of phospholipase C, which triggers the production of InsP3 and the opening of calcium channels. In sperm cells, this leads to an increase in mitochondrial calcium, which improves mitochondrial function and prevents apoptosis [51]. Furthermore, MI is thought to have a possible anti-oxidative effect, which may aid in the preservation of sperm function [52]. To investigate this, Mohammadi et al. performed a randomized prospective study, which evaluated the efficacy of incorporating MI in a freeze medium versus a control medium [53]. In this study, the number of ROS did not change between the groups; however, DNA fragmentation was significantly decreased in the MI group. Furthermore, in the MI-treated frozen/thawed samples, sperm progressive motility and normal morphology were increased versus in the control samples.

**Table 3:** Summary of studies of myo-inositol in male factor infertility.

Reference	Study Type	Number of Patients	Treatment Type and Length	Primary Findings
Calogero (2015) [44]	Double-blind randomized placebo-controlled study	194 patients with idiopathic infertility.	Study group: MI (2 g) + folic acid (200 µg) sachet twice daily. Control group: folic acid (200 µg) for 3 months.	Significant increases in acrosome-reacted spermatozoa, sperm concentration, total sperm count, and sperm progressive motility.
Rubino (2015) [47]	Prospective bicentric randomized study	500 MII sibling oocytes injected during 78 ICSI cycles. n = 262 study group; n = 238 placebo.	Spermatozoa were either treated with MI (2 mg/mL) or a placebo prior to in vitro culture.	Significant increase in fertilization rate and embryo quality.
Montanino Oliva (2016) [46]	Prospective longitudinal study	45 patients with asthenospermia and metabolic syndrome.	MI (1 g), L-carnitine (30 mg), L-arginine (30 mg), vitamin E (30 mg), selenium (55 µg), and 200 µg folic acid. Twice daily for 3 months.	Significant increase in testosterone, sperm concentration, motility, and normal morphology.
Mohammadi (2019) [53]	Randomized prospective study	Semen samples from 40 normozoospermic men.	Spermatozoa were either treated with MI (2 mg/mL) or a placebo prior to freezing.	Significant increase in progressive motility and normal morphology. After freezing, MI-treated samples showed reduced liquid peroxidation and DNA fragmentation.

MI: myo-inositol.

## 6. Conclusions

The use of MI has continued to expand, and it is no longer solely seen as an insulin sensitizer. The studies discussed within this article demonstrate the great potential for MI in both patients with and without PCOS seeking IVF care. While the literature does not provide a unified consensus, the use of MI prior to ovarian stimulation may positively affect gonadotropin use and duration, oocyte and embryo quality, fertilization, and clinical pregnancy rates. It should be noted that studies regarding the use of MI in IVF-ET are still limited, often with small sample size and performed in combination with other treatments such as folic acid, melatonin, or metformin. As such, larger RCTs are required to further probe the mechanism of action of MI in IVF. Furthermore, despite the varied studies regarding MI in IVF care, to the best of our knowledge, no large-scale meta-analyses have been conducted; such work would greatly help evaluate the true potential of MI in IVF.

## 7. Position Statement from the Experts Group on Inositol in Basic and Clinical Research and on PCOS (EGOI-PCOS), the Polish Society of Andrology, and the International Scientific Association for the Support and Development of Medical Technologies

It is the recommendation of the Experts Group on Inositol in Basic and Clinical Research and on PCOS (EGOI-PCOS), the Polish Society of Andrology, and the International Scientific Association for the Support and Development of Medical Technologies that MI be considered in pretreatment strategies prior to ovarian hyperstimulation with gonadotropins. The use of MI has been demonstrated to at the very least reduce gonadotropin use and duration in both non-PCOS and PCOS patient groups, thus reducing patient exposure to excessive levels of gonadotropin; in addition, it may potentially result in financial savings for ART procedures. Furthermore, the use of MI in IVF-ET may also increase oocyte and embryo quality, in addition to increasing fertilization rate and pregnancy outcomes. It should also be noted that MI has a well-established safety and tolerability profile, making it an ideal candidate for routine use, with minimal risk. In non-PCOS and PCOS populations, it is advised that MI (4 g) + folic acid (400 µg) supplementation be started three months prior to the start of COH. It is also recommended that treatment be continued throughout pregnancy, as MI can be beneficial in reducing the risk of gestational complications [54]. In PCOS populations with obesity, the use of MI and DCI in a 40:1 ratio may be considered; however, further study is required to establish the efficacy of this supplementation regime. Lastly, while MI may be effective in ICSI procedures for male factor infertility, more evidence is required. These recommendations are in line with the recently published NICE-adapted guidelines from the Italian Society of Human Reproduction, who stated “Consider prescribing MI prior to IVF because it can reduce the total dose of administered gonadotropins” [14].

## References

[1] Wyns C., Bergh C., Calhaz-Jorge C., De Geyter C., Kupka M.S., Motrenko T., Rugescu I., Smeenk J., Tandler-Schneider A., The European IVF-Monitoring Consortium (EIM) for the European Society of Human Reproduction and Embryology (ESHRE) (2020). ART in Europe, 2016: Results generated from European registries by ESHRE†. Hum. Reprod. Open.

[2] Zegers-Hochschild F., Dickens B.M., Dughman-Manzur S. (2014). Human Rights To In Vitro Fertilization. JBRA Assist. Reprod..

[3] Kawwass J.F., Penzias A.S., Adashi E.Y. (2021). Fertility-a human right worthy of mandated insurance coverage: The evolution, limitations, and future of access to care. Fertil. Steril..

[4] Ali M., Mathur R. (2023). Ovarian hyperstimulation syndrome: A review of recent practices. Obstet. Gynaecol. Reprod. Med..

[5] Sun B., Ma Y., Li L., Hu L., Wang F., Zhang Y., Dai S., Sun Y. (2020). Factors Associated with Ovarian Hyperstimulation Syndrome (OHSS) Severity in Women With Polycystic Ovary Syndrome Undergoing IVF/ICSI. Front. Endocrinol..

[6] Eapen A., Ryan G.L., Ten Eyck P., Van Voorhis B.J. (2020). Current evidence supporting a goal of singletons: A review of maternal and perinatal outcomes associated with twin versus singleton pregnancies after in vitro fertilization and intracytoplasmic sperm injection. Fertil. Steril..

[7] Tocariu R., Stan D., Mitroi R.F., Căldăraru D.E., Dinulescu A., Dobre C.E., Brătilă E. (2023). Incidence of complications among in vitro fertilization pregnancies. J. Med. Life.

[8] Dinicola S., Unfer V., Facchinetti F., Soulage C.O., Greene N.D., Bizzarri M., Laganà A.S., Chan S.Y., Bevilacqua A., Pkhaladze L. (2021). Inositols: From Established Knowledge to Novel Approaches. Int. J. Mol. Sci..

[9] Dinicola S., Minini M., Unfer V., Verna R., Cucina A., Bizzarri M. (2017). Nutritional and Acquired Deficiencies in Inositol Bioavailability. Correlations with Metabolic Disorders. Int. J. Mol. Sci..

[10] Merviel P., James P., Bouée S., Le Guillou M., Rince C., Nachtergaele C., Kerlan V. (2021). Impact of myo-inositol treatment in women with polycystic ovary syndrome in assisted reproductive technologies. Reprod. Health.

[11] Cabrera-Cruz H., Oróstica L., Plaza-Parrochia F., Torres-Pinto I., Romero C., Vega M. (2020). The insulin-sensitizing mechanism of myo-inositol is associated with AMPK activation and GLUT-4 expression in human endometrial cells exposed to a PCOS environment. Am. J. Physiol. Endocrinol. Metab..

[12] Laganà A.S., Garzon S., Casarin J., Franchi M., Ghezzi F. (2018). Inositol in Polycystic Ovary Syndrome: Restoring Fertility through a Pathophysiology-Based Approach. Trends Endocrinol. Metab..

[13] Buratini J., Dellaqua T.T., Dal Canto M., La Marca A., Carone D., Mignini Renzini M., Webb R. (2022). The putative roles of FSH and AMH in the regulation of oocyte developmental competence: From fertility prognosis to mechanisms underlying age-related subfertility. Hum. Reprod. Update.

[14] Palomba S., Viganò P., Chamayou S., Donarelli Z., Costantini M.P., Marci R., Piomboni P., Fino E., Montano L., Guglielmino A. (2024). Diagnosis and management of infertility: NICE-adapted guidelines from the Italian Society of Human Reproduction. Reprod. Biol. Endocrinol..

[15] Fitz V., Graca S., Mahalingaiah S., Liu J., Lai L., Butt A., Armour M., Rao V., Naidoo D., Maunder A. (2024). Inositol for Polycystic Ovary Syndrome: A Systematic Review and Meta-analysis to Inform the 2023 Update of the International Evidence-based PCOS Guidelines. J. Clin. Endocrinol. Metab..

[16] Carlomagno G., Unfer V. (2011). Inositol safety: Clinical evidences. Eur. Rev. Med. Pharmacol. Sci..

[17] Unfer V., Raffone E., Rizzo P., Buffo S. (2011). Effect of a supplementation with myo-inositol plus melatonin on oocyte quality in women who failed to conceive in previous in vitro fertilization cycles for poor oocyte quality: A prospective, longitudinal, cohort study. Gynecol. Endocrinol..

[18] Seyedoshohadaei F., Abbasi S., Rezaie M., Allahvaisi A., Jafar Rezaie M., Soufizadeh N., Rahmani K. (2022). Myo-inositol effect on pregnancy outcomes in infertile women undergoing in vitro fertilization/intracytoplasmic sperm injection: A double-blind RCT. Int. J. Reprod Biomed..

[19] Ferraretti A.P., La Marca A., Fauser B.C., Tarlatzis B., Nargund G., Gianaroli L. (2011). ESHRE consensus on the definition of ’poor response’ to ovarian stimulation for in vitro fertilization: The Bologna criteria. Hum. Reprod..

[20] Caprio F., D’Eufemia M.D., Trotta C., Campitiello M.R., Ianniello R., Mele D., Colacurci N. (2015). Myo-inositol therapy for poor-responders during IVF: A prospective controlled observational trial. J. Ovarian Res..

[21] Nazari L., Salehpour S., Hosseini S., Saharkhiz N., Azizi E., Hashemi T., Ghodssi-Ghassemabadi R. (2020). Effect of myo-inositol supplementation on ICSI outcomes among poor ovarian responder patients: A randomized controlled trial. J. Gynecol. Obstet. Hum. Reprod..

[22] Mohammadi S., Eini F., Bazarganipour F., Taghavi S.A., Kutenaee M.A. (2021). The effect of Myo-inositol on fertility rates in poor ovarian responder in women undergoing assisted reproductive technique: A randomized clinical trial. Reprod. Biol. Endocrinol..

[23] Dinicola S., Unfer V., Soulage C.O., Yap-Garcia M.I.M., Bevilacqua A., Benvenga S., Barbaro D., Wdowiak A., Nordio M., Dewailly D. (2024). D-chiro-inositol in clinical practice: A perspective from The Experts Group on Inositol in Basic and Clinical Research (EGOI). Gynecol. Obstet. Investig..

[24] Nordio M., Bezerra Espinola M.S., Bilotta G., Capoccia E., Montanino Oliva M. (2023). Long-Lasting Therapies with High Doses of D-chiro-inositol: The Downside. J. Clin. Med..

[25] Ravanos K., Monastra G., Pavlidou T., Goudakou M., Prapas N. (2017). Can high levels of D-chiro-inositol in follicular fluid exert detrimental effects on blastocyst quality?. Eur. Rev. Med. Pharmacol. Sci..

[26] Nasiri N., Eftekhari-Yazdi P. (2015). An overview of the available methods for morphological scoring of pre-implantation embryos in in vitro fertilization. Cell J..

[27] Melo A.S., Ferriani R.A., Navarro P.A. (2015). Treatment of infertility in women with polycystic ovary syndrome: Approach to clinical practice. Clinics.

[28] Raffone E., Rizzo P., Benedetto V. (2010). Insulin sensitiser agents alone and in co-treatment with r-FSH for ovulation induction in PCOS women. Gynecol. Endocrinol..

[29] Agrawal A., Mahey R., Kachhawa G., Khadgawat R., Vanamail P., Kriplani A. (2019). Comparison of metformin plus myoinositol vs metformin alone in PCOS women undergoing ovulation induction cycles: Randomized controlled trial. Gynecol. Endocrinol..

[30] Rajasekaran K., Malhotra N., Mahey R., Khadgawat R., Kalaivani M. (2022). Myoinositol versus metformin pretreatment in GnRH-antagonist cycle for women with PCOS undergoing IVF: A double-blinded randomized controlled study. Gynecol. Endocrinol..

[31] Emekçi Özay Ö., Özay A.C., Çağlıyan E., Okyay R.E., Gülekli B. (2017). Myo-inositol administration positively effects ovulation induction and intrauterine insemination in patients with polycystic ovary syndrome: A prospective, controlled, randomized trial. Gynecol. Endocrinol..

[32] Lesoine B., Regidor P.A. (2016). Prospective Randomized Study on the Influence of Myoinositol in PCOS Women Undergoing IVF in the Improvement of Oocyte Quality, Fertilization Rate, and Embryo Quality. Int. J. Endocrinol..

[33] Beresniak A., Russo M., Forte G., Laganà A.S., Oliva M.M., Aragona C., Chiantera V., Unfer V. (2023). A Markov-model simulation of IVF programs for PCOS patients indicates that coupling myo-Inositol with rFSH is cost-effective for the Italian Health System. Sci. Rep..

[34] Zheng X., Lin D., Zhang Y., Lin Y., Song J., Li S., Sun Y. (2017). Inositol supplement improves clinical pregnancy rate in infertile women undergoing ovulation induction for ICSI or IVF-ET. Medicine.

[35] Laganà A.S., Vitagliano A., Noventa M., Ambrosini G., D’Anna R. (2018). Myo-inositol supplementation reduces the amount of gonadotropins and length of ovarian stimulation in women undergoing IVF: A systematic review and meta-analysis of randomized controlled trials. Arch. Gynecol. Obstet..

[36] Roseff S., Montenegro M. (2020). Inositol Treatment for PCOS Should Be Science-Based and Not Arbitrary. Int. J. Endocrinol..

[37] Elnashar A.M. (2023). Update on obesity and assisted reproductive technology. Middle East Fertil. Soc. J..

[38] Colazingari S., Treglia M., Najjar R., Bevilacqua A. (2013). The combined therapy myo-inositol plus d-chiro-inositol, rather than d-chiro-inositol, is able to improve IVF outcomes: Results from a randomized controlled trial. Arch. Gynecol. Obstet..

[39] Agarwal A., Mulgund A., Hamada A., Chyatte M.R. (2015). A unique view on male infertility around the globe. Reprod. Biol. Endocrinol..

[40] Mazzilli R., Vaiarelli A., Dovere L., Cimadomo D., Ubaldi N., Ferrero S., Rienzi L., Lombardo F., Lenzi A., Tournaye H. (2022). Severe male factor in in vitro fertilization: Definition, prevalence, and treatment. An update. Asian J. Androl..

[41] Krzastek S.C., Farhi J., Gray M., Smith R.P. (2020). Impact of environmental toxin exposure on male fertility potential. Transl. Androl. Urol..

[42] Colone M., Marelli G., Unfer V., Bozzuto G., Molinari A., Stringaro A. (2010). Inositol activity in oligoasthenoteratospermia—An in vitro study. Eur. Rev. Med. Pharmacol. Sci..

[43] Ghasemi A., Amjadi F., Masoumeh Ghazi Mirsaeed S., Mohammad Beigi R., Ghasemi S., Moradi Y., Tahereh Ghazi Mirsaeed S. (2019). The effect of Myo-inositol on sperm parameters and pregnancy rate in oligoasthenospermic men treated with IUI: A randomized clinical trial. Int. J. Reprod. Biomed..

[44] Calogero A.E., Gullo G., La Vignera S., Condorelli R.A., Vaiarelli A. (2015). Myoinositol improves sperm parameters and serum reproductive hormones in patients with idiopathic infertility: A prospective double-blind randomized placebo-controlled study. Andrology.

[45] Jankowska K., Suszczewicz N., Rabijewski M., Dudek P., Zgliczyński W., Maksym R.B. (2022). Inhibin-B and FSH Are Good Indicators of Spermatogenesis but Not the Best Indicators of Fertility. Life.

[46] Montanino Oliva M., Minutolo E., Lippa A., Iaconianni P., Vaiarelli A. (2016). Effect of Myoinositol and Antioxidants on Sperm Quality in Men with Metabolic Syndrome. Int. J. Endocrinol..

[47] Rubino P., Palini S., Chigioni S., Carlomagno G., Quagliariello A., De Stefani S., Baglioni A., Bulletti C. (2015). Improving fertilization rate in ICSI cycles by adding myoinositol to the semen preparation procedures: A prospective, bicentric, randomized trial on sibling oocytes. J. Assist. Reprod. Genet..

[48] Borate G.M., Meshram A. (2022). Cryopreservation of Sperm: A Review. Cureus.

[49] Kopeika J., Thornhill A., Khalaf Y. (2015). The effect of cryopreservation on the genome of gametes and embryos: Principles of cryobiology and critical appraisal of the evidence. Hum. Reprod. Update.

[50] Walke G., Gaurkar S.S., Prasad R., Lohakare T., Wanjari M. (2023). The Impact of Oxidative Stress on Male Reproductive Function: Exploring the Role of Antioxidant Supplementation. Cureus.

[51] Condorelli R.A., La Vignera S., Di Bari F., Unfer V., Calogero A.E. (2011). Effects of myoinositol on sperm mitochondrial function in-vitro. Eur. Rev. Med. Pharmacol. Sci..

[52] Osman R., Lee S., Almubarak A., Han J.I., Yu I.J., Jeon Y. (2023). Antioxidant Effects of Myo-Inositol Improve the Function and Fertility of Cryopreserved Boar Semen. Antioxidants.

[53] Mohammadi F., Varanloo N., Heydari Nasrabadi M., Vatannejad A., Amjadi F.S., Javedani Masroor M., Bajelan L., Mehdizadeh M., Aflatoonian R., Zandieh Z. (2019). Supplementation of sperm freezing medium with myoinositol improve human sperm parameters and protects it against DNA fragmentation and apoptosis. Cell Tissue Bank.

[54] Greff D., Váncsa S., Váradi A., Szinte J., Park S., Hegyi P., Nyirády P., Ács N., Horváth E.M., Várbíró S. (2023). Myoinositols Prevent Gestational Diabetes Mellitus and Related Complications: A Systematic Review and Meta-Analysis of Randomized Controlled Trials. Nutrients.

